# The effect of *Trichoderma harzianum* agents on physiological-biochemical characteristics of cucumber and the control effect against Fusarium wilt

**DOI:** 10.1038/s41598-023-44296-z

**Published:** 2023-10-17

**Authors:** Hua Lian, Runzhe Li, Guangshu Ma, Zhenghan Zhao, Ting Zhang, Mei Li

**Affiliations:** 1 College of Horticulture and Landscape Architecture, Heilongjiang Bayi Agricultural University, Daqing, 163319 Heilongjiang China; 2 Institute of Plant Protection, Chinese Academy of Agricultural Sciences, Beijing, 100193 China; 3 Key Laboratory of Intergrated Pest Management in Crops, Ministry of Agriculture and Rural Affairs, Beijing, 100193 China

**Keywords:** Biophysics, Physiology, Plant sciences

## Abstract

At the seedling and adult plant phases, pot experiments were carried out to enhance the physiological-biochemical characteristics of cucumber, guarantee its high yield, and ensure its cultivation of quality. *Trichoderma harzianum* conidia agents at 10^4^, 10^5^, 10^6^, and 10^7^ cfu g^−1^ were applied in accordance with the application of Fusarium oxysporum powder at concentrations of 10^4^ cfu/g on the protective enzyme activity, physiological and biochemical indices, seedling quality, resilience to Fusarium wilt, quality, and yield traits. Fusarium oxysporum powder at 10^4^ cfu g^−1^ was used to treat CK1, while Fusarium oxysporum powder and *T. harzianum* conidia agents were not used to treat CK2. The results show that different *T. harzianum* agents improved the activities of superoxide dismutase (SOD), ascorbate peroxidase (APX), catalase (CAT), and peroxidase (POD) in cucumber seedlings, improved chlorophyll content, root activity, root-shoot ratio, and seedling strength index, and decreased malondialdehyde (MAD) content (P < 0.05). T3, a combination of 10^4^ cfu g^−1^ Fusarium oxysporum powder and 10^6^ cfu g^−1^ T*. harzianum* conidia agents, had the greatest promoting effect. The effects of different *T. harzianum* conidia agents and their application amounts on the control of cucumber Fusarium wilt were explored. T3 had the best promotion impact, and the control effect of cucumber Fusarium wilt at seedling stage and adult stage reached 83.98% and 70.08%, respectively. The quality index and yield formation of cucumber were also increased by several *T. harzianum* agents, with T3 having the strongest promotion effects. In comparison to CK1, the soluble sugar, Vc, soluble protein, and soluble solid contents of T3 cucumber fruit were 120.75%, 39.14%, 42.26%, and 11.64% higher (*P* < 0.05), respectively. In comparison to CK2, the soluble sugar, Vc, soluble protein, and soluble solid contents of T3 cucumber fruit were 66.06%, 24.28%, 36.15%, and 7.95% higher (*P* < 0.05), respectively. In comparison to CK1 and CK2, the yields of T3 cucumber were 50.19% and 35.86% higher, respectively. As a result, *T. harzianum* agents can enhance the physiological and biochemical traits of cucumber seedlings, raise the quality of cucumber seedlings, have a controlling impact on Fusarium wilt, and increase the yield and quality of cucumber fruit. The greatest effectiveness of T3 comes from its use. In this study, *Trichoderma harzianum* conidia agents demonstrated good impacts on cucumber yield formation and plant disease prevention, demonstrating their high potential as biocontrol agents.

## Introduction

Cucumber (*Cucumis sativus* L.) is one of the principal vegetable crops in China, with the largest planting area and the broadest range of cultivation^1^. According to estimates from the FAO (Food and Agricultural Organization of the United Nations), China will produce 1.27 million hm^2^ of cucumbers in 2020, whereas the rest of the world will produce 2.25 million hm^2^. The world produces 90.35 million tons of cucumbers annually, with China producing 73.36 million tons, or 81.2% of the total^2^. Additionally, China's cucumber planting area and yield, which make up roughly 6% of the nation's total vegetable planting area and almost 10% of the total production, are on the upswing. The main barrier to the production of facility cucumber in China has been the escalation of soil-borne diseases brought on by continuous cropping^3^, with cucumber wilt being one of the most significant diseases. This is because planting areas are growing year after year, especially in facility production. Cucumber Fusarium wilt is a soil-borne fungal disease caused by *Fusarium oxysporum* f. sp. *cucumerinum* Owen. All of the cucumber's growth stages are affected by the disease cycle. The incidence rate is typically 10%–30%, but in severe years, it can reach 50%. The yield loss ranges from 10 to 50%, and in certain cases, a crop may fail completely^4^. The main causes of the disease, which is becoming more and more serious, include long-term continuous cropping, declining soil fertility, and an imbalanced microbial flora^5^. One of the most challenging issues in cucumber cultivation is cucumber wilt. Chemical pesticides are regarded as the "sharp tool" for eradicating disease. Yet, the misuse of chemical pesticides leads to many hidden risks and lowers their efficiency in preventing disease. Biological control has gradually developed as a research hotspot for Fusarium wilt control due to its benefits of safety, friendliness, low cost, and widespread availability^6^. Under the criteria for effective and safe disease management, *Trichoderma* has gradually emerged as one of the most researched and used biological control fungi.

*Trichoderma* is a fungus that can be found in the Ascomycota subclass, the Sordreomycetes subclass, the Hyphomycetes subclass, the Hypocreomycetidae subclass, the Hyphomycetes order, the Sphaeriales order, or the Hyphomycetes family. *Trichoderma*, *Hypocrea*^7^. *Trichoderma* has the ability to stimulate plant development by secreting chemicals such as plant growth hormone, as demonstrated by Ahmad et al.^8^. *Trichoderma* has been shown in numerous studies to enhance plant growth, physiological metabolism, yield, and quality. For instance, Metwally et al.^9^ demonstrated that *Trichoderma aviride* and *Arbuscular mycorrhizal* fungi might boost onion morphological characteristics such as leaf area, stem length, root length, and pigment content in addition to fresh weight and dry weight. According to Zhang et al.^10^, *Trichoderma harzianum* may boost the cucumber's soluble sugar, soluble protein, chlorophyll, and root activity, as well as encourage the plant's development under salt stress. Although Estifanos et al.^11^ discovered that *Trichoderma* can boost cucumbers' output in addition to their plant height, When Carlos and José^12^ administered *Trichoderma polysporum* LCB50 to melon alone, the yield increased by 27%, and the control effect of melon Fusarium wilt reached 32.2%.

*Trichoderma* can increase plant resistance by secreting secondary metabolites and enzymes that break down cell walls, as demonstrated by Harman^13^. The defense enzyme system, which primarily consists of catalase (CAT), peroxidase (POD), superoxide dismutase (SOD), ascorbate peroxidase (APX), etc., primarily manifests plant disease resistance, and there is a slight positive correlation between the activity of defense enzymes and plant disease resistance^14^. When pathogenic bacteria infect plants, biocontrol microorganisms like *Trichoderma* can cause changes in the plant's disease-resistant defense enzymes and enhance the plant's ability to fend off infection. The defense response is closely related to the enhancement of various protective enzyme activities^15^. In addition, when the plant becomes contaminated, there will be significant electrolyte leakage in the cell. Malondialdehyde (MDA) is a key component of the plant's response to disease resistance and can be utilized as a biomarker of the degree of membrane lipid peroxidation^16^. Several studies have revealed that *Trichoderma* can enhance plant growth, disease resistance, and antioxidant system performance. For instance, Mohamed et al.^17^ investigated the interaction between *Trichoderma asperellum* T34 and Enterobacter cloacae PS14 to control potato bacterial wilt. The findings revealed that the two were more effective when applied together, increasing POD, lipoxygenase, and PPO activities and reducing the incidence of bacterial wilt by 10.7% to 26.5% in greenhouses and 26.6% to 36.6% in fields.

For more than 60 years, *Trichoderma* products have been utilized throughout the world. Between September 2022 and September 1983, when Cornell University produced the first *T. harzianum* biocontrol product, there would be 280 fungicides with *Trichoderma* as the active component, making up nearly 60% of the global market for biological fungicides. In the course of its life cycle, *Trichoderma* can produce mycelium, conidia, and chlamydospores. At present, there are more than 50 kinds of commercial *Trichoderma* preparations at home and abroad^18^, such as *T. harzianum* T22 strain in the United States and *T. harzianum* T39 strain in Israel; *Trichoderma* preparations Trichodry and Trichoflow in New Zealand; *Trichoderma* preparations Myc01 in Russia; *Trichoderma* YC458 from South Korea; and the mixed biocontrol agent TUSAL of *T. harzianum* and *Trichoderma viridis* in Spain^19^. They are either combined preparations of conidia and mycelium from *Trichoderma* or preparations of Trichoderma conidia. This is due to the difficulty in producing the *Trichoderma* chlamydospore preparation and the very tight artificial fermentation conditions, which restrict its production and utilization. Conidia preparation differs from chlamydospore preparation in that it has a short shelf life and a control effect that is easily influenced by environmental factors. *Trichoderma* conidia can, however, be produced in a variety of solid or liquid media under the right circumstances and are generally tolerant of a wide range of environmental conditions. Because of this, conidia preparations make up the majority of *Trichoderma* preparations utilized in manufacturing. To ensure high yield and high-quality plant production, it is crucial to keep developing *Trichoderma* conidia preparations with steady performance. *T. harzianum* is easier to colonize at plant roots than other microbial strains, and certain strains have rhizosphere ability, which allows them to grow on the root while they are still developing. *Trichoderma harzianum* grows more quickly and is very adaptable to challenging soil conditions. *Trichoderma harzianum* stands out among the diverse soil bacteria as a result. In the previous plate face-off test against Fusarium wilt of cucumber, *T. harzianum* 809, which was chosen for this study, had an inhibition rate of 85.16% and a field control effect of 65.48%. *T. harzianum* conidia 809 was chosen as the test strain in this investigation as a result. The physiological mechanisms of *Trichoderma harzianum* conidia 809 to enhance the resistance of cucumber to Fusarium wilt infection were thoroughly studied, as were the effects on cucumber seedling growth, physiological characteristics, yield, quality, the antioxidant system, and the control effect of Fusarium wilt. *Trichoderma* was employed to reduce cucumber Fusarium wilt under the conditions of "double reduction" of pesticides and fertilizers and "zero" pesticide growth. It can offer technical assistance for the planting of healthy, high-quality cucumbers and serve as a theoretical foundation for further studies into *Trichoderma*'s development and promotion.

## Materials and methods

### Materials

#### Test cucumber cultivar

Changchun Mici, a cucumber cultivar from Yuyuan Seed Corporation Ltd., Xintai, Shandong, China, was used in this study. Changchun Mici has a large planting area in China. The use of cucumber cultivars in the study complies with international, national, and institutional guidelines. The collection of plant material and the performance of experimental research on cucumber complied with the national guidelines of China.

#### Test medium

Refer to Li et al.^20^, PDA culture medium (200 g of potato, 20 g of glucose, 10 g of agar, 1 000 mL of distilled water), PD culture medium (200 g of potato, 20 g of glucose, 1 000 mL of distilled water), and Fusarium oxysporum selective medium (1.0 g of potassium dihydrogen phosphate, 0.5 g of potassium chloride, 0.5 g of magnesium sulfate, 0.01 g of ethylenediamine tetraacetic acid, 2.0 g of L-asparagine, 20.0 g of D-galactin, and 1,000 distilled water sterilized at 121 degrees Celsius for 20 min). Refer to Masunaka et al.^21^ for the composition of PDAM, the *Trichoderma* selective culture medium (200 g of peeled potato, 20 g of glucose, 20 g of agar, 0.3 g of chloramphenicol, 0.02 g of rose red, which is Bengal red, and 1 000 mL of distilled water).

#### Test strain

The tested strain of *Trichoderma harzianum* 809 and the pathogenic fungus *Fusarium oxysporum* sp. *cucumebrium* Owen were both donated by the *Trichoderma* research group at the Institute of Plant Protection, Chinese Academy of Agricultural Sciences, Beijing, China.

#### Test substrate material

Turf:vermiculite (volume ratio) was a 2:1 mixture utilized as the substrate material for the test. The substrate material's fundamental physicochemical characteristics were as follows: pH value of 6.92, organic matter content of 5.63%, total nitrogen content of 0.84%, alkali-hydrolyzed nitrogen content of 137.79 mg/kg, fast-acting phosphorus content of 130.29 mg/kg, and fast-acting potassium content of 198.63 mg/kg. The mixed substrate was screened to a thickness of 1 mm and sterilized in the oven for two hours at 160 °C. Following natural cooling, it continues to sterilize for another two hours at 160 °C before being chilled for use.

### Preparation of Fusarium oxysporum powder and Trichoderma spore powder

#### Preparation of Fusarium oxysporum powder

The pathogenic fungi that cause cucumber Fusarium wilt were grown on PDA culture media for 3 days in the dark at 28 °C after thoroughly cleaning the agar surface with distilled water. To manufacture the phytopathogenic fungi's spores, five samples with a 5 mm diameter were taken from the borders of each fungal colony. These samples were then infused with 100 mL of the PD liquid culture medium into 250 mL conical flasks. The flasks were cultured in a shaking incubator for 7 days at 28 °C (250 rpm). The mycelium was removed using double-layer gauze filtration, and the filtrate was centrifuged at 5000 rpm for 10 min. To make the pathogen spore powder, the precipitated spores were suspended in sterile water (equal to the fermentation liquid), 3% diatomite was added, the mixture was mixed in, it was filtered, and it was dried. To measure the pathogen spore content, the powder was diluted in a sterile water gradient, coated with Fusarium oxysporum selective media, and left for 1 h. The culture plate was then placed in a 28 °C incubator for 3–4 days. The amount of spores, 1.9 × 10^7^ cfu g^−1^, was estimated, the number of colonies was counted, and the applied dose was established in compliance with the test requirements.

#### Preparation of Trichoderma harzianum conidia powder

For an activation culture, *Trichoderma harzianum* 809 was grown on PDA culture media for three days at 28 °C in the dark. Five samples, each measuring 5 mm in diameter, were taken from the edges of each colony to prepare the Trichoderma spore suspension. These samples were then transferred to PDA culture medium and incubated for seven days at 28 °C in the dark. The spores were then harvested by washing them with distilled water. It is coated on the *Trichoderma* selective medium and inverted for 1–2 days at 25–28 °C after being diluted with sterile water gradient. Calculate the amount of *Trichoderma* conidia by counting the colonies. Barley grains were sterilized by soaking them in clean, room-temperature water for an entire night. The water was then removed, and 1 kg of fresh-keeping bags were filled with it. *Trichoderma harzianum* suspensions were added after chilling, and they were then cultured for two to three weeks at 25 °C. Rinse with sterile water and filter away the grain after the spore has grown too large. *Trichoderma harzianum* conidium powder was created by crushing, filtering, and drying the filtrate after 10% talcum powder was added. *Trichoderma harzianum* conidiomyces had a concentration of 2.4 × 10^9^ cfu g^−1^, and the administered dose was determined in accordance with the test specifications.

### Test methods

#### Seedling test

This experiment was carried out in a plastic greenhouse at the Heilongjiang Bayi Agricultural University's teaching base for the College of Horticulture and Landscape Architecture in China. Plastic seedling culture dishes (34.5 cm × 24 cm × 11 cm) containing the sterilized soil were then filled, and various concentrations of *Trichoderma harzianum* conidia were mixed in. Eighty cucumber seeds were put in each plate following germination treatment, and fifty seedlings of similar size were kept. After seeding, cucumbers were kept in a normal growth stage by being given 1000 mL of sterile water every two days.

In this study, there were 6 different treatments, each of which had 50 seedlings in 5 dishes. The experiment was carried out four times, selecting seedlings at random each time.

The treatment groups were as follows:

1. 10^4^ cfu g^−1^ Fusarium oxysporum powder and 10^4^ cfu g^−1^ *Trichoderma harzianum* conidia agent (T1)
2. 10^4^ cfu g^−1^ Fusarium oxysporum powder and 10^5^ cfu g^−1^ *Trichoderma harzianum* conidia agent (T2)
3. 10^4^ cfu g^−1^ Fusarium oxysporum powder and 10^6^ cfu g^−1^ *Trichoderma harzianum* conidia agent (T3)
4. 10^4^ cfu g^−1^ Fusarium oxysporum powder and 10^7^ cfu g^−1^ *Trichoderma harzianum* conidia agent (T4)
5. 10^4^ cfu g^−1^ Fusarium oxysporum powder only (CK1)
6. Neither Fusarium oxysporum powder nor *Trichoderma harzianum* conidia agent (CK2)

To measure the antioxidant enzymes, physiological indicators, and biochemical indicators of cucumber seedlings at 15 and 30 days after sowing, 40 plants were randomly chosen from each treatment (10 plants per replication). After 15 days of germination, the cucumber's ability to control disease was assessed. At 30 days after sowing, 40 plants were chosen from each treatment (10 plants per replication) in order to calculate the root-shoot ratio and strong seedling index, as well as seedling morphological indices and material accumulation indices for cucumber seedlings.

#### Adult plant stages test

The test substrate material was still chosen to be potting soil. Hebei Qianyuan Plastic Products Co., Ltd. manufactures the potted plastic bucket. The plastic bucket measures 30 cm in upper diameter, 27 cm in lower diameter, 20 L in volume, and 30 cm in height. The following ingredients were evenly distributed into each plastic bucket: 15 kg of potting soil (7 cm from the top edge); 1.5 g diamine phosphate; 4.5 g potassium sulfate; and 7.5 g urea. The fertilizers and soil were thoroughly combined, then compacted.

In this study, there were six different treatments, each with 20 buckets (set as a plot). The experimental environment was the same as in 4.3.1. The experiment was carried out four times with a random selection of all seedlings. The spacing between each replicate and the buckets inside the plot were both fixed at 70 cm.

The potted experiment was conducted on April 15, 2020, in a plastic greenhouse at the Heilongjiang Bayi Agricultural University, China, which serves as the college's teaching base. After receiving a germination treatment, the cucumber seeds were placed in buckets with 12 seeds each, and six seedlings of similar size were chosen. After seeding, sterile water was poured into the bucket every two days to maintain the soil's moisture and maintain the cucumber's regular growth. Two seedlings of comparable growth, two leaves, and one heart stage were chosen from the bucket. Field maintenance procedures included timely hanging vines, single vine rectification, and insect control.

To determine the plot yield throughout the course of the entire harvest, the cumulative sum of the fruits gathered was added up, and the theoretical cucumber yield per hectare was then converted to the plot area. Investigations into the incidence, disease index, and impact of preventive measures were done during the peak period of results (the fourth to tenth harvest periods). Four fruits per replication were randomly chosen from each treatment after the tenth harvest, resulting in a total of 16 fruits for each treatment. Mixed fruit samples were then utilized to calculate quality indices. From each treatment, 16 fruits (4 fruits per replicate) were.

randomly chosen, and the weight of each fruit was measured.

### Test indicators and methods

#### Determination of relevant indexes of the antioxidant enzyme system

The nitroblue tetrazole photochemical reduction technique was used to measure the activity of superoxide dismutase (SOD)^22^. The ascorbic acid technique was used to assess ascorbic acid peroxidase (APX) activity^23^. The UV absorption method was used to assess the catalase (CAT) activity^24^. The guaiacol technique was used to measure the peroxidase (POD) activity^25^.

#### Determination of physiological and biochemical indicators

The acetone-ethanol technique was used to determine the content of chlorophyll^26^. The naphthylamine oxidation method was used to assess the vitality of the roots^27^. Anthrone colorimetry was used to calculate the content of soluble sugar present^28^. Using the Coomassie Brilliant Blue G-250 staining procedure, the content of soluble proteins was identified^29^. Malondialdehyde (MDA) content was measured with thiobarbituric acid^30^. Sulfosalicylic acid and the acidic ninhydrin color technique were used to extract proline (Pro) content^31^.

#### Determination of morphological indicators

The distance between the stem base and its growth point on each seedling was measured with a ruler to calculate the plant height. A vernier caliper was used to measure the stem diameter 1 cm below the cotyledon region^32^.

#### Determination of material accumulation indicators

The plants were frequently rinsed in clean water and dried on absorbent paper thereafter. According to the plants' above-ground and subsurface components, the fresh weight of the plants was calculated. The fresh samples were then dried at 105 °C for 15 min and baked to a constant weight at 70 °C. An electronic scale with a resolution of 1/1000 was used to measure the dry weight of the above- and below-ground components^33^.

The following formula was used to determine the root-shoot ratio using Gou et al.'s technique^34^:

$${\text{Root}} - {\text{shoot ratio}}:{\text{ fresh weight of underground part}}/{\text{fresh weight of above}} - {\text{ground part}}.$$

The formula below was used to determine the strong seedling index according to Liu et al.'s method^35^:

$$\begin{aligned} & {\text{Strong seedling index}}: \, \left( {{\text{Stem diameter}}/{\text{Plant height }} + {\text{ underground dry weight }}/{\text{ above ground dry weight}}} \right) \\ & \quad \times {\text{ dry}} {\text{weight of whole plant}} \\ \end{aligned}.$$

#### Determination of disease resistance indicators

The incidence rate, disease index, and control effect are components of the disease resistance indices.

The percentage of diseased plants in each treatment compared to the total number of plants under investigation 15 days after sowing was the incidence rate at the seedling stage. After the tenth cucumber harvest, the incidence rate at adult plant stages was calculated as the ratio of the number of diseased plants in each treatment to the total number of plants under investigation.

Elagamey et al.^36^ evaluated the severity of cucumber Fusarium wilt, while Zhang et al.^37^ employed the disease index.

Grade 0: no symptoms;

Grade 1: The yellowing or wilting area of true leaves and cotyledons does not exceed 50% of the total area;

Grade 2: The yellowing or wilting area of true leaves and cotyledons exceeds 50% of the total area;

Grade 3: Leaves are wilted or dead, with only growing points surviving;

Grade 4: The entire plant is severely wilted or dead.

$${\text{Disease index}} = \sum \left( {{\text{Number of disease grades}} \times {\text{Grade number}}} \right)/\left( {{\text{total plant number}} \times {\text{the highest gradenumber}}} \right) \times {1}00\%.$$

$${\text{Control effect }}\left( \% \right) = \left( {{\text{Disease index in the control group}} - {\text{Disease index in the treated group}}} \right)/{\text{Disease index in the control group}} \times {1}00.$$

#### Determination of fruit quality indicators

The contents of soluble protein and sugar refer to the procedure in (3). Iodometry was used to estimate the vitamin C concentration^38^. With the use of an Abel refractometer, the content of soluble solids was measured.

#### Determination of yield indicators

The TB-4002 analytical scale from Beixiaogan Yaguang Medical Electronics Company Ltd. was used to weigh a single cucumber with a precision of 0.01 g. By growing 45,000 cucumber plants per hectare, the hypothetical yield of cucumbers was computed.

### Statistical analysis

Data statistics and variance analysis were performed using DPS 7.05 software, and Duncan's novel complicated range approach was utilized to assess multiple comparisons among various treatments (*P* < 0.05). For mapping, 2019's original program was used.

## Results

### The effect of T. harzianum agents on the antioxidant enzyme activities of cucumber seedlings leaves

**Figure 1:**
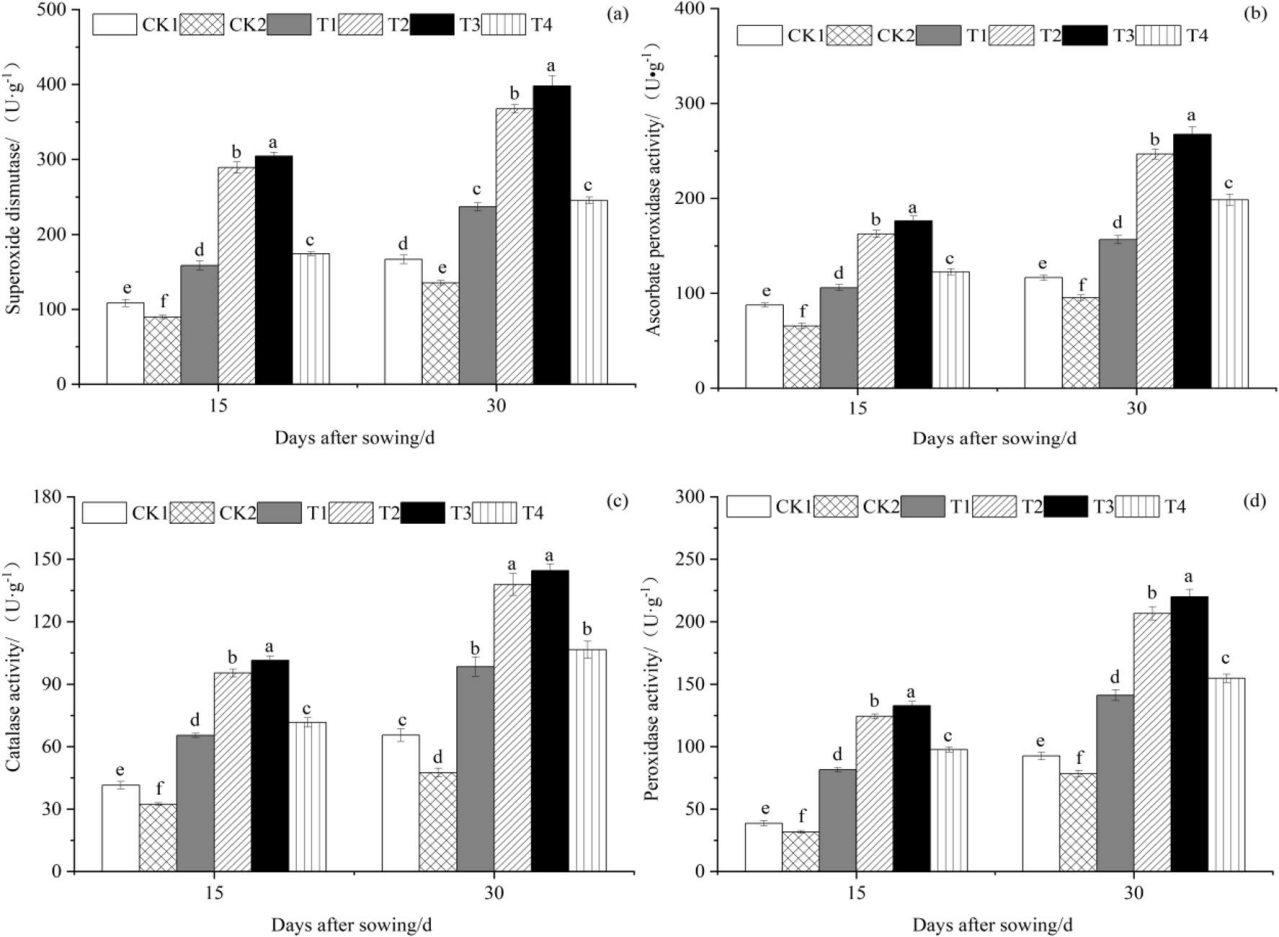
The effect of *T. harzianum* agents on the antioxidant enzyme activities of cucumber seedlings leaves. Error bars show SD, and different letters indicate significant differences at 5% (*p* < 0.05) level.

The antioxidant enzyme activities of cucumber seedling leaves were assessed at 15 and 30 days after sowing under various *T. harzianum* concentrations, respectively. Figure 1a,b,c,d display the results.

Figure 1a illustrates that at 15 and 30 days after sowing, SOD activity of cucumber leaves treated with various concentrations of *T. harzianum* was higher than that of CK1, which is 10^4^ cfu g^−1^ Fusarium oxysporum powder alone, and CK2, which is not treated with Fusarium oxysporum powder and *Trichoderma harzianum* conidia agents. SOD activity of cucumber leaves increased initially but then decreased as treatment concentration increased. The highest levels of SOD activity, 304.3983 U·g^−1^ and 398.0620 U·g^−1^, were found in cucumber leaves treated with T3 (10^4^ cfu g^−1^ Fusarium oxysporum powder and 10^6^ cfu g^−1^ T*. harzianum* conidia agents). The SOD activity of T3 leaves was noticeably greater than that of other treatments 15 days after sowing. The concentrations of T1 (10^4^ cfu g^−1^ Fusarium oxysporum powder and 10^4^ cfu g^−1^ T*. harzianum* conidia agents), T2 (10^4^ cfu g^−1^ Fusarium oxysporum powder and 10^5^ cfu g^−1^ T*. harzianum* conidia agents), T3, and T4 (10^4^ cfu g^−1^ Fusarium oxysporum powder and 10^7^ cfu g^−1^ T*. harzianum* conidia agents) were significantly (*P* < 0.05) higher than those of CK1 and CK2, which increased by 6.19%, 166.78%, 180.82%, 60.53%, and 76.74%, or 222.54%, 239.52%, and 94.08%, respectively. CK1 had a huge advantage over CK2, which grew by 20.90%. Compared to other treatments, T3 leaves had considerably increased SOD activity 30 days after sowing. T1, T2, T3, and T4 all had much higher values than CK1 and CK2, whose values rose (*P* < 0.05) by 42.16%, 120.52%, 138.86%, 47.34%, and 75.04%, or 171.51%, 194.09%, and 81.41%, respectively. CK1 was much greater than CK2, which rose by 23.12%.

According to Fig. 1b, similar to SOD activity, APX activity in cucumber leaves changed regularly in response to *T. harzianum* treatments. The APX activity of cucumber leaves under different treatments of *T. harzianum* was higher than that of CK1 and CK2 at 15 and 30 days after sowing, and the APX activity of cucumber leaves initially increased and subsequently dropped with the increase in *T. harzianum* treatment concentration. Cucumber leaves treated with T3 had the highest APX activity, with values of 176.4657 U·g^−1^ and 267.6110 U·g^−1^, respectively. The APX activity of T3 leaves was noticeably (*P* < 0.05) greater than that of other treatments 15 days after sowing. T1, T2, T3, and T4 all had much higher values than CK1 and CK2, whose values rose by 20.96%, 85.37%, 101.34%, 40.01%, and 61.46%, or 147.43%, 168.75%, and 86.89%, respectively. CK1 had a huge advantage over CK2, which grew by 33.48%. The APX activity of T3 leaves was substantially (*P* < 0.05) higher at 30 days after sowing compared to other treatments. T1, T2, T3, and T4 all had considerably higher values than CK1 and CK2, whose values rose by 34.38%, 111.72%, 129.66%, 70.46%, and 63.89%, 158.21%, 180.09%, and 107.89%, respectively. CK1 had a considerable advantage over CK2, which grew by 21.96%.

As shown in Fig. 1c, CAT activity of cucumber leaves under different treatments of *T. harzianum* was higher than that of CK1 and CK2 at 15 and 30 days after sowing, and CAT activity of cucumber leaves first increased and then decreased with the increase in *T. harzianum* treatment concentration. Cucumber leaves treated with T3 had the greatest CAT activity, at 101.4867 U·g^−1^ and 144.5463 U·g^−1^, respectively. The CAT activity of T3 leaves was noticeably (*P* < 0.05) greater than that of other treatments 15 days after sowing. T1, T2, T3, and T4 were all considerably (*P* < 0.05) higher than CK1 and CK2, whose values increased by 57.62%, 129.81%, 144.51%, 72.56%, and 102.56%, 195.33%, 214.22%, and 121.76%, respectively. CK1 had a considerable advantage over CK2, which grew by 28.51%. The CAT activity of T3 leaves was noticeably greater than that of other treatments 30 days after sowing. T1, T2, T3, and T4 were all considerably (*P* < 0.05) higher than CK1 and CK2, which increased by 50.25%, 110.53%, 120.64%, 62.61%, and 107.35%, 190.53%, 204.49%, and 124.40%, respectively. CK1 was noticeably greater than CK2, which rose by 38.00%.

Figure 1d illustrate that 15 and 30 days after sowing, the POD activity of cucumber leaves under various *Trichoderma harzianum* treatment concentrations was higher than that of CK1 and CK2, and the POD activity of cucumber leaves first increased and then declined with the increase in *T. harzianum* treatment concentration. Cucumber leaves treated with T3 had the greatest POD activity, with values of 132.7757 U·g^−1^and 220.0253 U·g^−1^, respectively. The POD activity of T3 leaves was noticeably (*P* < 0.05) greater than that of other treatments 15 days after sowing. T1, T2, T3, and T4 levels were significantly (*P* < 0.05) higher than CK1 and CK2, which increased by 111.33%, 222.59%, 244.62%, 153.32%, and 156.98%, respectively, and by 292.27%, 319.06%, and 208.05%. CK1 increased significantly more than CK2, which increased 21.60%. The POD activity of T3 leaves was significantly (*P* < 0.05) higher than that of other treatments 30 days after sowing. T1, T2, T3, and T4 increased by 52.70%, 123.54%, 138.01%, 67.37%, and 80.23%, 163.83%, 180.91%, and 97.55%, respectively, compared to CK1 and CK2. The increase in CK1 was significantly greater than the increase in CK2, which was 18.03%.

### Effect of T. harzianum agents on the physiological and biochemical indexes of cucumber seedling

#### Effect of T. harzianum agents on the chlorophyll content and root activity of cucumber seedling

**Figure 2:**
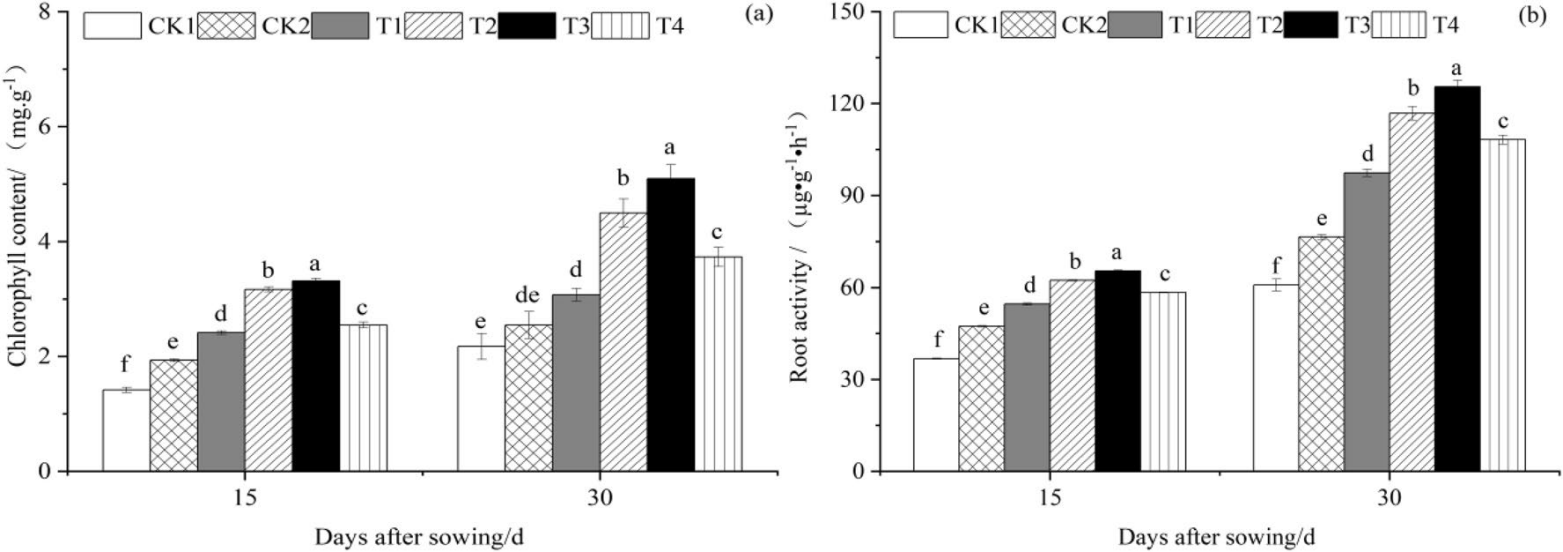
The effect of *T. harzianum* agents on the chlorophyll content and root activity of cucumber seedlings. Error bars show SD, and different letters indicate significant differences at 5% (*p* < 0.05) level.

Chlorophyll content and root activity in cucumber seedlings were measured 15 and 30 days after sowing, respectively, using different *T. harzianum* treatments. Figure 2a,b show the results.

#### Effect of T. harzianum agents on the soluble sugar and soluble protein content of cucumber seedling leaves

As illustrated in Fig. 2a, at 15 and 30 days after sowing, the chlorophyll content of cucumber seedling leaves under various *T. harzianum* treatments was higher than that of CK1 and CK2, and the chlorophyll content of cucumber seedlings first increased and then decreased as *T. harzianum* treatment concentration increased. Cucumber seedlings treated with T3 had the highest chlorophyll content, at 3.3160 mg·g^−1^ and 5.0957 mg·g^−1^. The chlorophyll content of T3 cucumber seedling leaves at 15 days after sowing was substantially higher than that of other treatments. T1, T2, T3, and T4 had significantly (*P* < 0.05) higher values than CK1 and CK2, whose values rose by 70.30%, 123.57%, 134.07%, 79.71%, and 24.88%, 63.94%, 71.64%, and 31.78%, respectively. CK1 had a huge advantage over CK2, which grew by 36.37%. The chlorophyll content of T3 cucumber seedling leaves at 30 days after sowing was substantially (*P* < 0.05) higher than that of other treatments. T1, T2, T3, and T4 were all noticeably higher than CK1 and CK2, whose values rose by 41.39%, 106.82%, 134.43%, 71.73%, and 20.82%, 76.72%, 100.30%, and 46.74%, respectively. Between CK2 and CK1, there was no discernible change.

As shown in Fig. 2b, the root activity in cucumber seedlings under various *T. harzianum* treatments was higher than that of CK1 and CK2 at 15 and 30 days after sowing, and the root activity in cucumber seedlings first increased and then decreased with the increase in *T. harzianum* treatment concentration. Root activity in cucumber seedlings treated with T3 was the highest at 65.4043 µg g^−1^ h^−1^ and 125.4790 µg g^−1^ h^−1^, respectively. The root activity in cucumber seedlings treated with T3 was noticeably higher than that of other treatments at 15 days following sowing. T1, T2, T3, and T4 had significantly (*P* < 0.05) higher values than CK1 and CK2, whose values rose by 48.61%, 69.59%, 78.00%, 58.90%, and 15.34%, 31.63%, 38.15%, and 23.33%, respectively. CK1 had a huge advantage over CK2, which grew by 28.84%. Cucumber seedlings treated with T3 had much more root activity than other treatments at 30 days after sowing. T1, T2, T3, and T4 had significantly (*P* < 0.05) higher values than CK1 and CK2, whose values rose by 60.24%, 92.17%, 106.41%, 78.03%, and 27.36%, 52.75%, 64.06%, and 41.50%, respectively. CK1 had a huge advantage over CK2, which grew by 25.81%.

**Figure 3:**
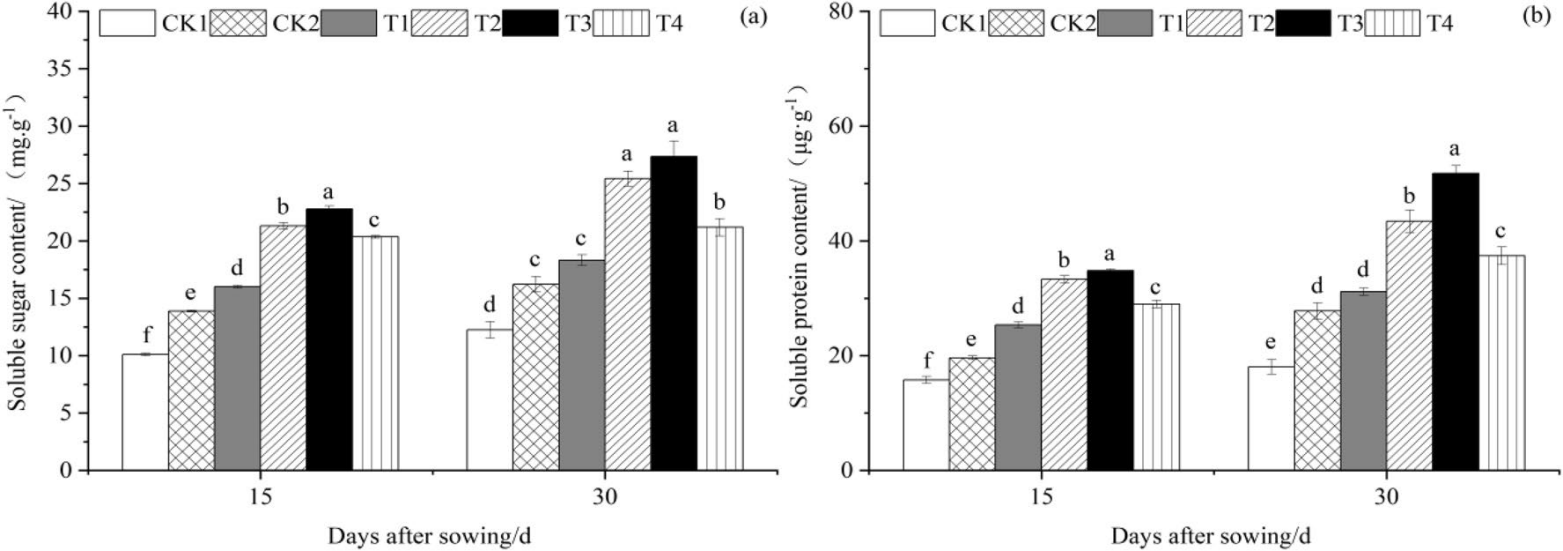
The effect of *T. harzianum* agents on the soluble sugar and soluble protein content of cucumber seedlings leaves. Error bars show SD, and different letters indicate significant differences at 5% (*p* < 0.05) level.

According to Fig. 3a, at 15 and 30 days after sowing, the soluble sugar content in cucumber seedling leaves under various *T. harzianum* treatment concentrations was higher than that of CK1 and CK2 and increased and then declined with the concentration of *T. harzianum* treatment. Cucumber seedling leaves treated with T3 had the highest soluble sugar content at 22,7827 mg g^−1^ and 27,3503 mg g^−1^, respectively. The soluble sugar concentration in cucumber seedling leaves of T3 was substantially higher than that of other treatments 15 days after sowing. T1, T2, T3, and T4 were all significantly (*P* < 0.05) higher than CK1 and CK2, which increased by 58.40%, 110.95%, 125.46%, 101.38%, and 15.28%, 53.52%, 64.08%, and 46.56%, respectively. CK1 was much greater than CK2, which rose by 37.41%. The soluble sugar content of cucumber seedling leaves from T3 was higher than that of other treatments at 30 days after sowing. T1, T2, T3, and T4 were greater (*P* < 0.05) than CK1 and CK2, which increased by 49.62%, 107.64%, 123.28%, 73.00%, and 13.00%, 56.82%, 68.63%, and 30.66%, respectively. CK1 was much greater than CK2, which rose by 32.41%.

As shown in Fig. 3b, soluble protein content in cucumber seedling leaves under different treatments of *T. harzianum* was higher than that of CK1 and CK2 at 15 and 30 days after sowing, and soluble protein content in cucumber seedling leaves first increased and then decreased with the increase in *T. harzianum* treatment concentration. Soluble protein content in cucumber seedling leaves treated with T3 was the highest at 34.8337 µg g^−1^ and 51.7420 µg g^−1^, respectively. The soluble protein content of cucumber seedling leaves from T3 was substantially (*P* < 0.05) higher than that of other treatments 15 days after sowing. T1, T2, T3, and T4 were all noticeably higher than CK1 and CK2, whose values climbed by 60.76%, 111.44%, 120.92%, 83.88%, and 29.28%, 70.04%, 77.67%, and 47.88%, respectively. The difference between CK1 and CK2, which increased by 24.35%, was notable. The soluble protein content of cucumber seedling leaves from the T3 treatment was higher at 30 days after sowing than it was for the other treatments. T1, T2, T3, and T4 were all higher (*P* < 0.05) than CK1 and CK2, whose values rose by 72.55%, 140.62%, 186.63%, 107.18%, and 12.11%, 56.34%, 86.22%, and 34.60%, respectively. CK1 rose considerably more than CK2, which rose by 53.91%.

#### Effect of T. harzianum agents on the malondialdehyde (MDA) and proline (Pro) content of cucumber leaves

**Figure 4:**
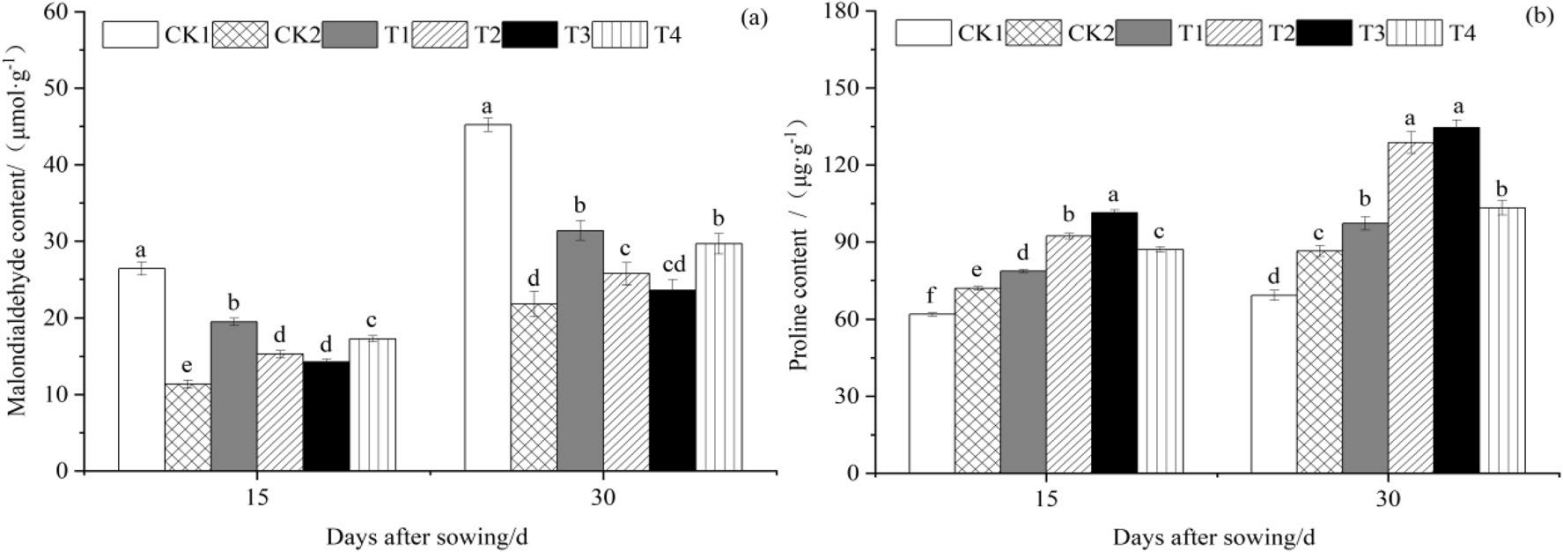
The effect of *T. harzianum* agents on the malondialdehyde and proline content of cucumber seedlings leaves. Error bars show SD, and different letters indicate significant differences at 5% (*p* < 0.05) level.

As seen in Fig. 4a, at 15 and 30 days after planting, malondialdehyde content in cucumber seedling leaves first reduced and subsequently increased as *T. harzianum* treatment concentration increased. At 15 and 30 days after sowing, malondialdehyde content in cucumber seedling leaves treated with CK1 was the highest, measuring 26.3990 mol/g and 45.2037 mol/g, and malondialdehyde content in CK2-treated cucumber seedling leaves was the lowest, measuring 11.3713 μmol g^−1^ and 21.8167 μmol g^−1^. The malondialdehyde level in the leaves of CK1 was significantly higher than that of other treatments at 15 days after sowing, and in comparison to CK1, the malondialdehyde content in the leaves of CK2, T1, T2, T3, and T4 decreased (*P* < 0.05) by 56.93%, 26.10%, 42.05%, 46.00%, and 34.52%, respectively. Compared to CK2, T1, T2, T3, and T4 had respective increases of 71.57%, 34.54%, 25.36%, and 52.01%, respectively. Malondialdehyde content in the leaves of CK1 was significantly (*P* < 0.05) higher than that of the other treatments at 30 days after sowing, while that of CK2, T1, T2, T3, and T4 was significantly lower than that of CK1 by 51.74%, 30.58%, 43.05%, 47.68%, and 34.39%, respectively. T1, T2, T3, and T4 all rose in comparison to CK2 by 43.83%, 18.00%, 8.40%, and.

35.93%, respectively.

Figure 4b demonstrates that at 15 and 30 days after sowing, proline content in cucumber seedling leaves under various *T. harzianum* treatment concentrations was higher than that of CK1 and CK2. Proline content in cucumber seedling leaves first increased and then decreased with the increase in *T. harzianum* treatment concentration. The highest proline concentrations were found in cucumber seedling leaves treated with T3, at 101.404 μg g^−1^ and 134.6023 μg g^−1^, respectively. The proline content in cucumber seedling leaves from T3 was substantially higher than that of other treatments 15 days after sowing. T1, T2, T3, and T4 were all significantly (*P* < 0.05) higher than CK1 and CK2, which increased by 26.92%, 48.98%, 63.66%, 40.48%, and 9.09%, 28.06%, 40.68%, and 20.75%, respectively. CK1 was much greater than CK2, which rose by 16.34%. The proline content of cucumber seedling leaves from T3 was higher than that of other treatments at 30 days after sowing. T1, T2, T3, and T4 were higher (*P* < 0.05) than CK1 and CK2, whose values rose by 40.19%, 85.58%, 94.05%, 48.97%, and 12.49%, 48.90%, 55.70%, and 19.53%, respectively. CK1 was much greater than CK2, which rose by 24.63%.

### Effect of T. harzianum agents on the root-shoot ratio and strong seedling index of cucumber

**Figure 5:**
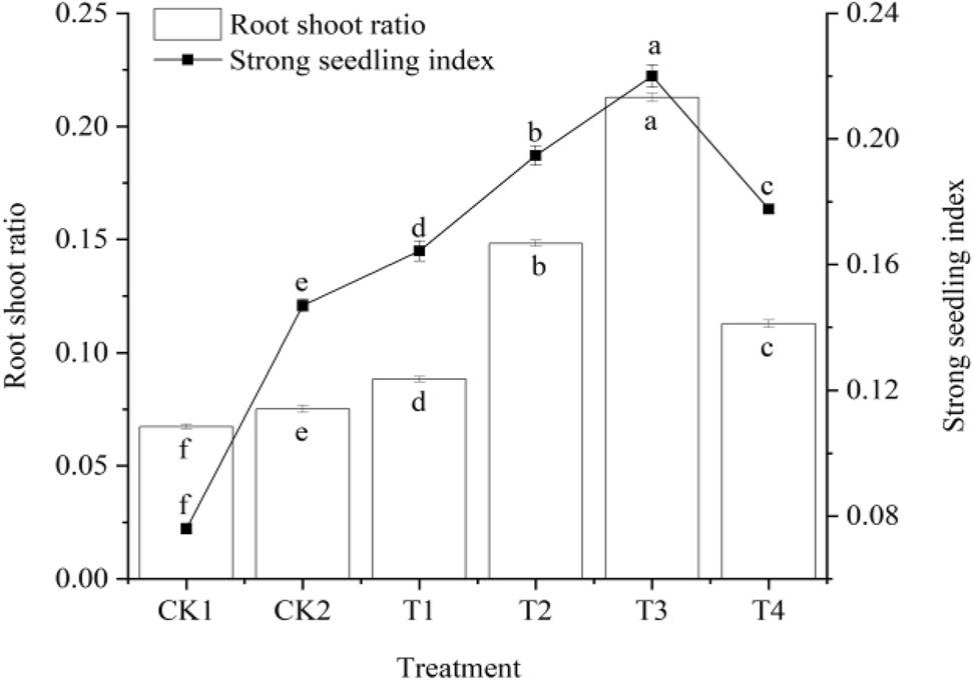
The effect of *T. harzianum* agents on the root-shoot ratio and strong seedling index of cucumber. Error bars show SD, and different letters indicate significant differences at 5% (*p* < 0.05) level.

The root-shoot ratio and strong seedling index were calculated at 30 days after sowing using the morphological indices of plant height and stem diameter as well as substance accumulation indices of fresh weight and dry weight in the aboveground and underground parts of cucumber seedlings, respectively. The outcomes are displayed in Fig. 5.

The root-shoot ratio and strong seedling index of cucumber seedlings increased at first and gradually declined with the increase in *T. harzianum* treatment concentration, as shown in Fig. 5. These traits were greater in *T. harzianum* treatments than in CK1 and CK2 treatments. Cucumber seedlings treated with T3 had the highest root-to-shoot ratio and strongest seedling index, at 0.2127 and 0.2200, respectively. Cucumber seedlings treated with T3 had a much higher root-to-shoot ratio than those receiving other treatments. T1, T2, T3, and T4 were all significantly (*P* < 0.05) higher than CK1 and CK2, which increased by 31.16%, 120.03%, 215.58%, 67.21%, and 17.55%, respectively, and by 97.21%, 182.85%, and 49.87%. CK1 was considerably greater than CK2, which increased by 11.57%. Cucumber seedlings treated with T3 had a considerably greater strong seedling index than those treated with other treatments. The values of T1, T2, T3, and T4 were much greater than those of CK1 and CK2, which increased by 116.18%, 156.18%, 189.47%, 133.82%, and 11.77%, respectively, and by 32.45%, 49.66%, and 20.88%. CK1 was substantially higher than CK2, which rose by 93.42%.

### Effect of Trichoderma harzianum agents against cucumber Fusarium wilt

#### Effect of Trichoderma harzianum agents against cucumber Fusarium wilt in the seedling stage

Using various concentrations of *T. harzianum* treatment, the incidence ratio, disease index, and control efficacy of cucumber seedlings were evaluated 15 days after sowing. The outcomes are shown in Table 1.

**Table 1:** Effect of *Trichoderma harzianum* agents against cucumber *Fusarium* wilt in the seedling stage.

Treatment	Incidence ratio/%	Disease index	Control efficacy/%
CK1	100.00 ± 0.32 a	51.61 ± 4.61 a	–
CK2	0	–	–
T1	18.85 ± 1.27 b	19.67 ± 0.84 b	61.89 ± 1.32 c
T2	7.46 ± 0.74 c	12.19 ± 0.71 c	76.38 ± 3.22 b
T3	6.32 ± 0.25 c	8.27 ± 0.73 d	83.98 ± 2.16 a
T4	15.68 ± 0.9 b	17.76 ± 0.81 b	65.59 ± 4.36 c

Values show the mean values ± SD, and different letters in the same column indicate significant at 5% (*p* < 0.05) level.

*T. harzianum* had a considerably reduced incidence ratio and disease index when treated at various concentrations other than CK1. In contrast to T1, T2, T3, and T4, which grew by 430.50%, 1240.48%, 1482.28%, 537.76%, and 162.38%, 323.38%, and 190.60%, respectively, the incidence ratio and disease index treated with CK1 were greater. Cucumber seedlings treated with T3 had the lowest incidence ratio and disease index, at 6.32% and 8.27, respectively. Cucumber seedlings treated with T3 had the highest control efficacy, at 83.98%. Cucumber seedlings treated with T3 had much greater control efficacy than those treated with T2, T4, or T1, which rose by 9.95%, 28.04%, and 35.69%, respectively.

#### Effect of T. harzianum agents against cucumber Fusarium wilts at the adult plant stage

After the tenth harvest of cucumber fruits, the incidence ratio, disease index, and control efficacy of cucumber at the adult plant stage were examined under various concentrations of *T. harzianum* treatment, respectively. The results are reported in Table 2. Under varied *T. harzianum* treatment concentrations, the incidence ratio and disease index were significantly lower than CK1. An increase of 39.51%, 125.46%, 170.81%, 62.64%, and 81.77%, 206.43%, 234.21%, and 105.18%, respectively, was seen in the incidence ratio and illness index treated with CK1 compared to T1, T2, T3, and T4. At only 31.62% and 17.54, respectively, the incidence ratio and disease index of cucumber seedlings treated with T3 were the lowest. The control efficacy of T3 and T2, though there was no statistically significant difference between them, were both substantially better than T1 and T4, which increased by 55.80%, 36.71%, and 49.78%, 31.43%, respectively.

**Table 2:** Effect of *Trichoderma harzianum* agents against cucumber *Fusarium* wilts at the adult plant stage.

Treatment	Incidence ratio/%	Disease index	Control efficacy/%
CK1	85.63 ± 6.98 a	58.62 ± 3.87 a	–
CK2	0	–	–
T1	61.38 ± 4.62 b	32.25 ± 1.32 b	44.98 ± 1.32 c
T2	37.98 ± 3.11 d	19.13 ± 0.98 c	67.37 ± 3.22 a
T3	31.62 ± 1.87 d	17.54 ± 0.55 c	70.08 ± 2.16 a
T4	52.65 ± 3.62 c	28.57 ± 0.63 b	51.26 ± 4.36 b

Values show the mean values ± SD, and different letters in the same column indicate significant at 5% (*p* < 0.05) level.

### Effect of T. harzianum agents on the quality and yield of cucumber

The effects of *T. harzianum* on the fruit quality and yield of cucumber were shown in Table 3. The highest amounts of soluble sugar, Vc, soluble protein, soluble solids, and yield were found in T3 cucumbers, with values of 29.36 mg g^−1^, 162.35 mg kg^−1^, 9.19 g kg^−1^, 4.89%, and 119,412.00 kg hm^−1^, respectively. The soluble sugar content and vitamin C content of T3 cucumber fruit were significantly (*P* < 0.05) higher than those of CK1, CK2, T1, T2, and T4, which increased by 120.75%, 66.06%, 49.57%, 14.55%, and 23.05%, and 39.14%, 24.28%, 18.88%, 5.79%, and 8.73%, respectively; CK2 was noticeably (*P* < 0.05)greater than CK1, which increased by 32.93% and 11.96%, respectively. Both T3 and T2 cucumber fruits had significantly.

**Table 3:** Effect of *T. harzianum* agents on the quality and yield of cucumber.

Treatment	Soluble sugar content/(mg·g^−1^)	Vitamin C content/(mg·kg^−1^)	Soluble protein content/(g·kg^−1^)	Soluble solid ontent /%	Yield/(kg·hm^−2^)
CK1	13.31 ± 0.45 e	116.68 ± 3.62 e	6.46 ± 0.02 e	4.38 ± 0.02 d	79,506.00 ± 24.96 e
CK2	17.68 ± 0.89 d	130.63 ± 1.95 d	6.75 ± 0.04 d	4.53 ± 0.03 c	87,894.00 ± 27.66 d
T1	19.63 ± 0.77 c	136.57 ± 8.32 c	8.41 ± 0.02 c	4.71 ± 0.02 b	98,856.00 ± 28.65 c
T2	25.63 ± 0.87 b	153.46 ± 6.35 b	9.08 ± 0.03 a	4.84 ± 0.03 a	113,341.50 ± 20.05 b
T3	29.36 ± 1.05 a	162.35 ± 4.22 a	9.19 ± 0.06 a	4.89 ± 0.07 a	119,412.00 ± 19.84 a
T4	23.86 ± 0.84 b	149.32 ± 4.06 b	8.74 ± 0.04 b	4.75 ± 0.06 b	109,669.50 ± 16.36 b

Values show the mean values ± SD, and different letters in the same column indicate significant at 5% (*p* < 0.05) level.

(*P* < 0.05) higher soluble protein contents than CK1, CK2, T1, and T4, which increased by 42.26%, 36.15%, 9.27%, 5.15%, and 40.56%, 34.52%, 7.97%, and 3.89%, respectively. CK2 was significantly (*P* < 0.05) higher than CK1, which increased by 4.49%. However, there was no statistically significant difference between the soluble protein contents of T3 and T2 cucumber fruits. While there was no discernible difference between the soluble solid contents of T3 and T2 cucumber fruits, both were significantly (*P* < 0.05) higher than those of CK1, CK2, T1, and T4, which increased by 11.64%, 7.95%, 3.82%, 2.95%, and 10.50%, 6.84%, 2.76%, and 1.89%, respectively; CK2 was noticeably higher than CK1, which increased by 32.93% and 11.96%, respectively. Compared to CK1, CK2, T1, T2, and T4, the yield of T3 cucumber rose by 50.19%, 35.86%, 20.79%, 5.36%, and 8.88%, respectively.

## Discussion

A popular biocontrol fungus for plant diseases called *Trichoderma* spp. has a wide range of antagonistic effects on fungi that cause plant diseases. Examples include *Rhizoctonia solani*, *Phytophthora* spp., *Pythium* spp., *Fusarium* spp., and others^39^. There have been numerous research reports on the use of biocontrol agents to control diseases such as *Trichoderma*. For instance, Mao Tingting and Jiang Xuanli^40^ investigated the *Trichoderma hamatum* strain MHT1134's control effects on Fusarium wilt in continuous pepper cropping fields. The results revealed that the MHT1134 control effects on pepper wilt after application for 1 year and 2 years were 63.03% and 70.21%, respectively. Yi Zhang et al.^41^ investigated the control effect of various *Trichoderma asperellum* application rates on watermelon Fusarium wilt, and the findings revealed that 10^5^ cfu/g *T. asperellum* M45a granules had an improved control effect on Fusarium wilt during the blooming period (up to 67.44%) in soils subjected to five years of continuous cropping with watermelon. The T3 treatment, which in this investigation involved applying 10^6^ cfu g^−1^ T*. harzianum* conidia agents, provided the best control effects on cucumber Fusarium wilt at the seedling and adult stages, with control effects of 83.98% and 70.08%, respectively. It is now widely accepted that *Trichoderma* has biological control effects through fungal parasitism and the induction of plant defense system resistance^42^.

*Trichoderma* is an important plant rhizosphere-promoting fungus (PGPF) in addition to having significant biological control significance. *Trichoderma* can help plants absorb minerals more quickly, assist in the control of plant growth, development, and carbohydrate metabolism, increase the growth of the host^43^, enhance the physiological metabolism of plants, and improve plant production and quality^44^. According to An-le HE et al.^45^, applying 2 g·pot^−1^ of *T. asperellum* GDFS1009 granules had the best control impact on stalk rot at the seedling stage (up to 53.7%), while the average plant height (up to 38.2 cm) and fresh weight (up to 1.89 g) were also noticeably enhanced. According to Zaw and Matsumoto^46^, *Trichoderma viride* TV 911 promoted the growth of Japanese mustard, tomato, and radish. The plant heights of mustard and tomato increased by 16.22% and 50.26%, respectively, while the fresh branch and root weights of radish increased by 23.83% and 58.86%, respectively. This is due to the fact that *Trichoderma* alters the physiological and biochemical metabolic processes of plants^47^, consequently enhancing their physiological metabolism^48^. Vij et al.^49^ investigated the effects of PGPR and bio-control agents on cabbage (*Brassica oleracea* L.) plants and demonstrated that these agents can significantly enhance seedling quality, growth, yield, and yield-related metrics over time. The increased percentages of seedling emergence, seedling height, and seedling stem diameter, the highest being 12%, 56%, and 57%, in T3 compared with the control treatment, further supported the combined effectiveness of the 5% *Trichoderma viride* and the 5% *Pseudomonas fluorescens* treatments before transplanting (T3). The seedling vigor index, seedling fresh weight, seedling dry weight, and chlorophyll content were the measurements where T3 significantly increased by 89%, 143%, 171%, and 26%, respectively, compared to the control. In addition, after receiving treatments, there were increases in the head weights and head yield of T3 of 40% and 37%, respectively, in comparison to the control. *T. harzianum* biofertilizer was investigated by Liu et al.^50^ for its effects on the growth, yield, and quality of Radix bupleuri and microbial responses. The findings revealed that *T. harzianum* biofertilizer promoted the growth of Bupleurum chinense and increased the yield and quality of radix bupleuri. Bupleurum chinense's stem length (100.38 cm), thickness (1.34 cm), root thickness (1.01 m), and total plant weight (8.66 g) all increased significantly when fertilized with *T. harzianum* biofertilizer in comparison to the control, increasing by respective amounts of 20.52%, 21.82%, 38.36%, and 126.70%. In contrast, *T. harzianum* biofertilizer considerably enhanced the levels of saikosaponins A (0.67%), C (0.65%), and D (0.71%) in radix bupleuri, increasing their concentrations by 8.06%, 47.73%, and 9.23%, respectively, in comparison to the control. Cucumber seedlings treated with T3 in this study had higher chlorophyll content, root activity, root-shoot ratio, and seedling strength index at 30 days after sowing than those treated with CK1, which is Fusarium oxysporum powder at 10^4^ cfu g^−1^ concentration alone. These differences were 134.43%, 106.41%, 215.58%, and 189.47%, respectively. Cucumber seedlings treated with T3 had higher levels of chlorophyll, root activity, root-to-shoot ratio, and seedling strength index than those treated with CK2, which had neither Trichoderma agent nor Fusarium oxysporum powder. These differences were 100.30%, 64.06%, 182.85%, and 49.66%, respectively. *T. harzianum can* also increase cucumber fruit yield and quality, with T3 being the best. In comparison to CK1, T3 cucumber fruit had soluble sugar, Vc, soluble protein, and soluble solid contents that were, respectively, 120.75%, 39.14%, 42.26%, and 11.64% greater. In comparison to CK2, T3 cucumber fruit had soluble sugar, Vc, soluble protein, and soluble solid contents that were, respectively, 66.06%, 24.28%, 36.15%, 7.95%, and higher than those of CK2. In comparison to CK1 and CK2, the yield of T3 cucumbers was 50.19% and 35.66% greater, respectively. These outcomes matched those of Vinale et al.^51^, who discovered that tomato and rape seedlings can develop more quickly and produce more when exposed to plant-like auxins that were extracted from *Trichoderma koningii* and *Trichoderma harzianum*.

Proline, soluble proteins, soluble sugars, and other compounds are examples of cell osmotic regulating chemicals that help keep cells balanced^52^. Proline content changes in plants can be used to determine how resistant plants are to stress circumstances because stress can greatly increase a plant's proline content^53^. The buildup of soluble protein can increase a plant's tolerance to stress and stop water loss from the cells. Furthermore, soluble proteins can function as the enzymes needed for a number of physiological metabolic activities. In order to activate the antioxidant system and protect themselves from damage, plants will also consume a significant number of soluble proteins in unfavorable conditions^54^. These osmoregulatory substances can be used as nutrients to support plant growth on the one hand, and on the other hand, they can act as significant osmotic regulatory substances in cells, controlling cell osmotic balance, improving cell structural stability, and lowering oxygen free radical production in tissues^55^. In this study, cucumber seedlings treated with T3 had soluble sugar, soluble protein, and proline concentrations in their leaves at 30 days after sowing that were, respectively, 123.28%, 186.63%, and 94.05% greater than those treated with CK1. Cucumber seedlings treated with T3 had higher concentrations of soluble sugar, soluble protein, and proline than those treated with CK2 by 68.63%, 86.22%, and 55.70%, respectively. In order to enhance the amount of soluble carbohydrates and proteins in plants, *Trichoderma* can create biologically regulating compounds such as gibberellin, growth hormone, and others^56^. The capacity of cells to hold water can therefore be improved by increasing these osmoregulation contents, which can also stabilize protein structure and lower the risk of cell death in stressed plants^57^.

During the interaction between *Trichoderma* and plants, *Trichoderma* induces plants to secrete secondary metabolites such as superoxide dismutase (SOD), ascorbic acid peroxidase (APX), catalase (CAT), peroxidase (POD), and polyphenol oxidase (PPO), and affects the content of physiological substances such as malondialdehyde (MDA). Both pathogen inhibition and systemic resistance induction in plants are possible with these compounds^48^. The primary byproducts of antioxidant enzymes and membrane lipid peroxidation have been linked in numerous earlier investigations to plant disease resistance. Cong et al.^58^, for instance, looked at the possibility of using mixed-culture fermentation (MCF) of Rhizopus nigricans and *Trichoderma pseudokoningii* to manage Fusarium oxysporum f.sp. cucumerinum in cucumber. Increased PAL, SOD, and POD defense enzyme activity in the pot experiment suggested that treatment with mixed broth might boost plant-induced resistance. The relative control impact on cucumber Fusarium wilt in the field trial was 76.5% after two years of treatment with the mixed broth. Boakye et al.^59^ investigated how *Trichoderma longibrachiatum* strains (TL6 and TL13) affected the growth-promoting potentials of snow pea seedlings and the ability to prevent root rot brought on by Fusarium solani (FSH) and Fusarium avenaceum (FAH). According to the findings, TL13 and TL6 in combination with FSH and FAH significantly lessened disease severity compared to controls by 86.6%, 81.6%, 57.60%, and 60.90%, respectively. However, the MDA and H_2_O_2_ content were reduced by 75.6%, 76.8%, 70.0%, and 76.4%, respectively, in contrast to the controls when FSH and FAH were coupled with TL6 and TL13. Moreover, compared to controls, the combinations TL6 + FSH and TL6 + FAH enhanced the activities of superoxide dismutase (SOD), peroxidase (POD), and catalase (CAT) by 60.5%, 64.7%, and 60.3%, 60.0%, 64.9%, and 56.6%, respectively. The combined effects of TL13 + FSH and TL13 + FAH elevated SOD, POD, and CAT activity by 69.7%, 68.6%, and 65.6%, and 70.10%, 69.5%, and 65.8%, respectively, in comparison to the controls. According to the findings, pretreating snow pea seeds with TL6 and TL13 promotes the growth of snow pea seedlings, inhibits FSH and FAH root rot, boosts antioxidant enzyme activity, and activates plant defense mechanisms. The findings of this study demonstrated that at 15 and 30 days after cucumber sowing, *T. harzianum* conidia agents had an impact on the activities of CAT, POD, SOD, APX, PPO, and malondialdehyde concentrations in cucumber seedling leaves. Moreover, compared to 15 days after cucumber seeding, the activities of CAT, POD, SOD, APX, PPO, and MDA levels rose at 30 days. The activity of protecting enzymes was significantly impacted by T3 30 days after cucumber sowing. SOD, APX, CAT, POD, and PPO activities were, respectively, 138.86%, 129.66%, 120.64%, 138.01%, and 253.87% greater in the leaves of T3 cucumber seedlings than those of CK1. SOD, APX, CAT, POD, and PPO activities were 194.09%, 180.09%, 204.49%, 180.91%, and 426.05% greater in the leaves of T3 cucumber seedlings than those of CK2, respectively. At the same time, 30 days after cucumber sowing, the MDA level in the leaves of T3 cucumber seedlings was 8.40% greater than that of CK2, and it was 47.68% lower than that of CK1. This could be because *T. harzianum*, which interacts with cucumber, induces protective enzymes such as CAT, POD, SOD, APX, and PPO in cucumber plants under pathogen infection, removes reactive oxygen species (ROS) in plants^60^, reduces MDA content^61^, and reduces cell membrane damage caused by membrane lipid peroxidation under disease stress.

## Conclusion

*Trichoderma harzianum* conidia agents prevent *Fusarium oxysporum* infection, enhance the physiological and biochemical functions of cucumber plants, and increase the protective enzyme activities. As a result, they improve cucumber seedling quality, increase the plants' resistance to Fusarium wilt, and increase cucumber yield and quality. The study's findings can offer technical assistance for *T. harzianum* agent development and use, as well as high yield and high-quality cucumber production, with promising future applications.

## Data Availability

The data generated and analysed in this study are available from the corresponding authors on reasonable request.
